# Effects of temperature, humidity, and diurnal temperature range on influenza incidence in a temperate region

**DOI:** 10.1111/irv.12682

**Published:** 2019-10-21

**Authors:** Ji‐Eun Park, Woo‐Sik Son, Yeonhee Ryu, Soo Beom Choi, Okyu Kwon, Insung Ahn

**Affiliations:** ^1^ Korea Institute of Oriental Medicine Daejeon Korea; ^2^ Center for Convergent Research of Emerging Virus Infection Korea Research Institute of Chemical Technology Daejeon Korea; ^3^ National Institute for Mathematical Science Daejeon Korea; ^4^ Biomedical Prediction Technology Laboratory Korea Institute of Science and Technology Information Daejeon Korea; ^5^ Department of Data‐centric Problem Solving Research Korea Institute of Science and Technology Information Daejeon Korea

**Keywords:** diurnal temperature range, humidity, influenza, temperature

## Abstract

**Background:**

The effect of temperature and humidity on the incidence of influenza may differ by climate region. In addition, the effect of diurnal temperature range on influenza incidence is unclear, according to previous study findings.

**Objectives:**

The aim of this study was to analyze the effects of temperature, humidity, and diurnal temperature range on the incidence of influenza in Seoul, Republic of Korea, which is located in a temperate region.

**Methods:**

We used Korean National Health insurance data to assess the weekly influenza incidence between 2010 and 2016, and used meteorological data from Seoul. To investigate the effect of temperature, relative humidity, and diurnal temperature range levels on influenza incidence, we used a distributed lag non‐linear model.

**Results:**

The risk of influenza incidence was significantly increased with low daily temperatures of 0‐5°C and low (30%–40%) or high (70%) relative humidity. We found a positive significant association between diurnal temperature range and influenza incidence in this study.

**Conclusions:**

Influenza incidence increased with low temperature and low/high humidity in a temperate region. Influenza incidence also increased with high diurnal temperature range, after considering temperature and humidity.

## 1 INTRODUCTION

Influenza places a great burden on public health and the economy.^1^ Various factors affect influenza transmission, including household structure,^2^ individual behavior,^3^ and climate variables.^4^ Previous studies have reported that environmental factors such as temperature, humidity, and precipitation can affect influenza transmission.^5^ Humidity and temperature conditions contribute to the seasonality of influenza outbreaks^6^; two types of environmental conditions are associated with influenza outbreaks: “cold‐dry” and “humid‐rainy.”^7^

Multiple studies have measured the association between low temperatures and influenza infection.^8^, ^9^ Several studies have reported the significant effect of temperature on influenza.^10^, ^11^ For example, Jaakkola et al^8^ showed that a 1°C decrease in temperature increased the estimated risk of influenza occurrence by 11% among military conscripts in Finland. However, other studies have found no significant association between temperature and influenza incidence.^9^

The relationship between humidity and influenza activity is unclear. Shaman et al^12^ showed that low absolute humidity drives seasonal variations in influenza transmission. The same researchers also reported that influenza activity modulated by seasonal changes in absolute humidity was consistent with the timing of pandemic influenza outbreaks.^13^, ^14^ A study based in the United States investigating the effect of humidity and temperature on influenza mortality reported that low absolute humidity was associated with increases in influenza mortality.^15^ However, one Japanese study reported that the number of patients with influenza increased significantly during times of high relative humidity.^16^ In addition, several studies did not find a significant association between humidity and influenza activity.^10^, ^17^

The association of temperature or humidity with influenza may differ by region. For example, Soebiyanto et al^18^ reported that seasonal influenza showed a positive association with humidity in El Salvador and Panama but a negative association in Guatemala, which is a similarly temperate region. Another study investigating the relationship between weather factors and influenza epidemics reported different exposure‐response relationships by region.^19^ Additional studies assessing the effect of weather on influenza in various regions are needed.

Previous works have reported that the diurnal temperature range (DTR) is a risk factor for mortality,^20^ including respiratory‐related mortality,^21^ cardiovascular mortality,^22^, ^23^ and asthma.^24^ Although several studies have reported the effect of DTR on influenza hospitalization^25^ or seasonal influenza,^26^ research assessing the effect of DTR on influenza is scarce and the findings controversial.

In this study, we aimed to assess the effect of temperature, humidity, and DTR on the incidence of influenza in Seoul, Republic of Korea, which is located in a temperate region.

## 2 METHODS

### 2.1 Data collection

We extracted the data of patients diagnosed with influenza between 2010 and 2016 from the database of the Korea National Health Insurance Service (NHIS‐2018‐1‐075). This database includes information of the obligatory national health insurance system, and beneficiaries include nearly the entire Korean population.^27^

Patients are diagnosed when they visit primary clinics or are admitted to hospitals. We extracted data from patients with disease codes J09 (influenza derived from a specific influenza virus strain), J10 (influenza derived from the influenza virus, strain not identified), and J11 (influenza, virus not identified), according to the Korea Informative Classification of Disease (KOICD).^28^

In this study, we used influenza incidence in Seoul, the capital of the Republic of Korea. Nearly 10 out of 50 million Koreans reside in Seoul.^29^ As daily incidence data are not provided in the Korea National Health Insurance Service to protect the personal information of patients, we used weekly influenza incidence data in this study.

Previous studies have shown a relationship between influenza and air pollution. In a Taiwan study, influenza among adults and older people was affected by ambient fine particles.^30^ A study in China showed that increased air pollutant concentrations were associated with an increase in cases of influenza‐like illness.^31^ A study by Nakao et al^32^ using Korean data reported that air pollution causes respiratory symptoms. Therefore, we included air pollution data as a covariate in this study.

Temperature, humidity, and air pollution data were obtained from the Korea Meteorological Administration Web site.^13^ As Korean meteorological stations only provide data of relative humidity without absolute humidity, relative humidity was used for the present analysis. Seoul meteorological stations provide data of daily temperature and relative humidity. Air pollution data were obtained from Air Quality Information of the Seoul Metropolitan Government.^33^ Air pollutants included in the analysis were ozone (O_3_), particulate matter <10 µm in diameter (PM_10_), nitrogen dioxide (NO_2_), and sulfur dioxide (SO_2_). Levels of these pollutants were obtained from the same source. For weekly meteorological data, mean data values in each week were used.

### 2.2 Statistical analysis

Associations between temperature, humidity, and air pollutants and the incidence of influenza were assessed using Spearman's correlation analysis. To investigate the non‐linear and delayed effects of meteorological factors on influenza incidence, a distributed lag non‐linear model was used. The effects of daily temperature and humidity were assessed using 5 *df* natural cubic spline for mean temperature and 1 *df* natural cubic spline for lag up to 2 weeks. The effect of DTR was assumed to be linear, based on previous studies.^20^ We used lags up to 2 weeks for temperature/humidity^10^, ^17^, ^34^ and DTR.^20^, ^35^ PM_10_, O_3_, and NO_2_ were controlled using 3 *df* natural cubic spline.^35^

$$Y_{t}∼\text{quasiPoisson}\;\left(\mu_{t}\right),\;t=1,\ldots T$$

$$\log\;\left(\mu_{t}\right)=\beta_{0}+\text{ns}\;\left(\text{temperature}\right)+\text{ns}\;\left(\text{humidity}\right)+\text{ns}\;\left(\text{TIME,}\;df=7\;\text{per year}\right)+\text{COVs}$$

where *Y_t_* denotes the influenza incidence count on day *t*, *μ_t_* is the expected influenza incidence on day *t*, and ns (temperature), ns (humidity), and ns (DTR) indicate a natural cubic spline of ambient temperature, relative humidity, and DTR on day *t*, respectively. COVs are potential confounders including ozone, NO_2_, SO_2_, and PM_10_. After comparison of Poisson, quasi‐Poisson, and negative binomial models, the quasi‐Poisson model was selected based on the residual and assumption tests. The quasi‐Poisson likelihood model was used to consider overdispersion. First, we investigated the effect of temperature and humidity. Subsequently, the effect of DTR was assessed, controlling for temperature and humidity. We reported the relative risk (RR) with 95% confidence intervals (CIs) of temperature, humidity, and change in DTR on influenza incidence with 10°C, 60%, and 8°C as reference values, respectively, which are close to the mean values. Residuals were evaluated to assess adequacy of the model.

For all statistical tests, two‐tailed *P* < .05 was considered statistically significant. The dlnm package in R software version 3.4.4 (The R Project for Statistical Computing, Vienna, Austria) was used to fit all the models. All analyses were conducted in 2019. For sensitivity analysis, we changed the degrees of freedom for time to control for season, and maximum lag for temperature, humidity, and DTR.

## 3 RESULTS

### 3.1 Influenza incidence and weather

Over the length of the observation period (January 3, 2010 to December 31, 2016), the mean weekly incidence of influenza was 3462.5 cases (highest: 100 253 cases). Temperatures ranged from −14.5°C to 31.8°C, and humidity ranged from 23.4% to 94.1%. Average temperature and humidity during this period were 13.0°C and 60.6%, respectively. The DTR was 1.4‐15.6°C with an average 8.4°C. Mean concentrations in Seoul of PM_10_, O_3_, NO_2_, and SO_2_ were 44.5 μg/m^3^, 24.5 ppb, 27.6 ppb, and 5.0 ppb (Table 1).

**Table 1:** Descriptive statistics of weekly influenza incidence, temperature, humidity, and air pollutant levels in Seoul, Republic of Korea (2010‐2016)

		Mean (SD)	Minimum	Q1	Q3	Maximum
Cases diagnosed/week (N)		3462.5 (8548.5)	200	422	2439	100 253
Weather	Temperature (°C)	13.0 (10.8)	−14.5	4.1	23.0	31.8
	Humidity (%)	60.6 (15.1)	23.4	48.5	72.8	94.1
	DTR (°C)	8.4 (2.9)	1.4	6.3	10.4	15.6
Air pollutant	PM10 (µg/m^3^)	44.5 (28.8)	4.7	26.3	54.0	248.5
	Ozone (ppb)	24.5 (12.6)	2.9	15.3	33.2	71.1
	NO_2_ (ppb)	27.6 (11.4)	9.0	18.9	35.0	62.1
	SO_2_ (ppb)	5.0 (1.6)	2.2	3.9	5.8	12.5

Note

Number of weeks is 365. There were no missing data.

Abbreviations: DTR, diurnal temperature range; NO_2,_ nitrogen dioxide; PM_10_, particulate matter <10 µm in diameter; SD, standard deviation; SO_2,_ sulfur dioxide.

**Figure 1:**
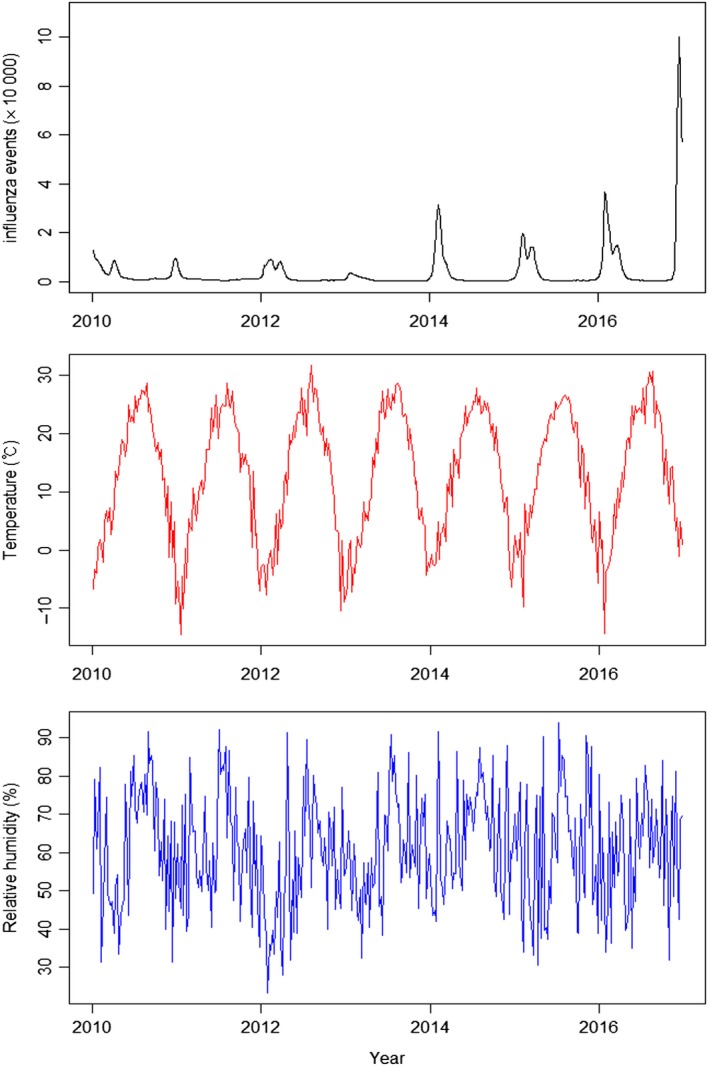
Trends of influenza incidence, temperature, and humidity during 2010‐2016 in Seoul, Republic of Korea

Figure 1 shows the trends of daily incidence for influenza, temperature, and humidity. Whereas one peak in influenza outbreaks can be seen in 2011, 2013, and 2014, two peaks are visible in other years. The incidence of influenza was higher during winter months (December‐February) than summer months. Temperature and humidity were highest during June to August, which is the summer season in Korea (Figure 1).

#### 3.1.1 Correlation between influenza incidence and meteorological factors

Influenza incidence was negatively associated with temperature (*r *= −.70) and humidity (*r *= −.36). A positive association with influenza incidence was observed for PM_10_ (*r* = .38), NO_2_ (*r* = .38), and SO_2_ (*r* = .39), and weak negative associations were observed for O_3_ (*r* = −.20) (Table 2).

**Table 2:** Spearman's correlation between meteorological factors and air pollutant levels and influenza incidence

	Influenza	Temperature	Humidity	DTR	PM10	Ozone	NO_2_	SO_2_
Influenza incidence	1							
Temperature	−0.70***	1						
Humidity	−0.36***	0.39***	1					
DTR	0.11*	0.02	−0.58***	1				
PM_10_	0.38***	−0.27***	−0.24***	0.26***	1			
Ozone	−0.20***	0.44***	−0.18***	0.32***	0.07	1		
NO_2_	0.38***	−0.40***	−0.17	0.27***	0.60***	−0.42***	1	
SO_2_	0.39***	−0.50***	−0.35***	0.27***	0.68***	−0.13*	0.67***	1

Abbreviations: DTR, diurnal temperature range; NO_2_, nitrogen dioxide; PM_10_, particulate matter <10 µm in diameter; SO_2_, sulfur dioxide.

* *P* < .05.

*** *P* < .001.

#### 3.1.2 Effects of temperature, humidity, and DTR on influenza incidence

**Figure 2:**
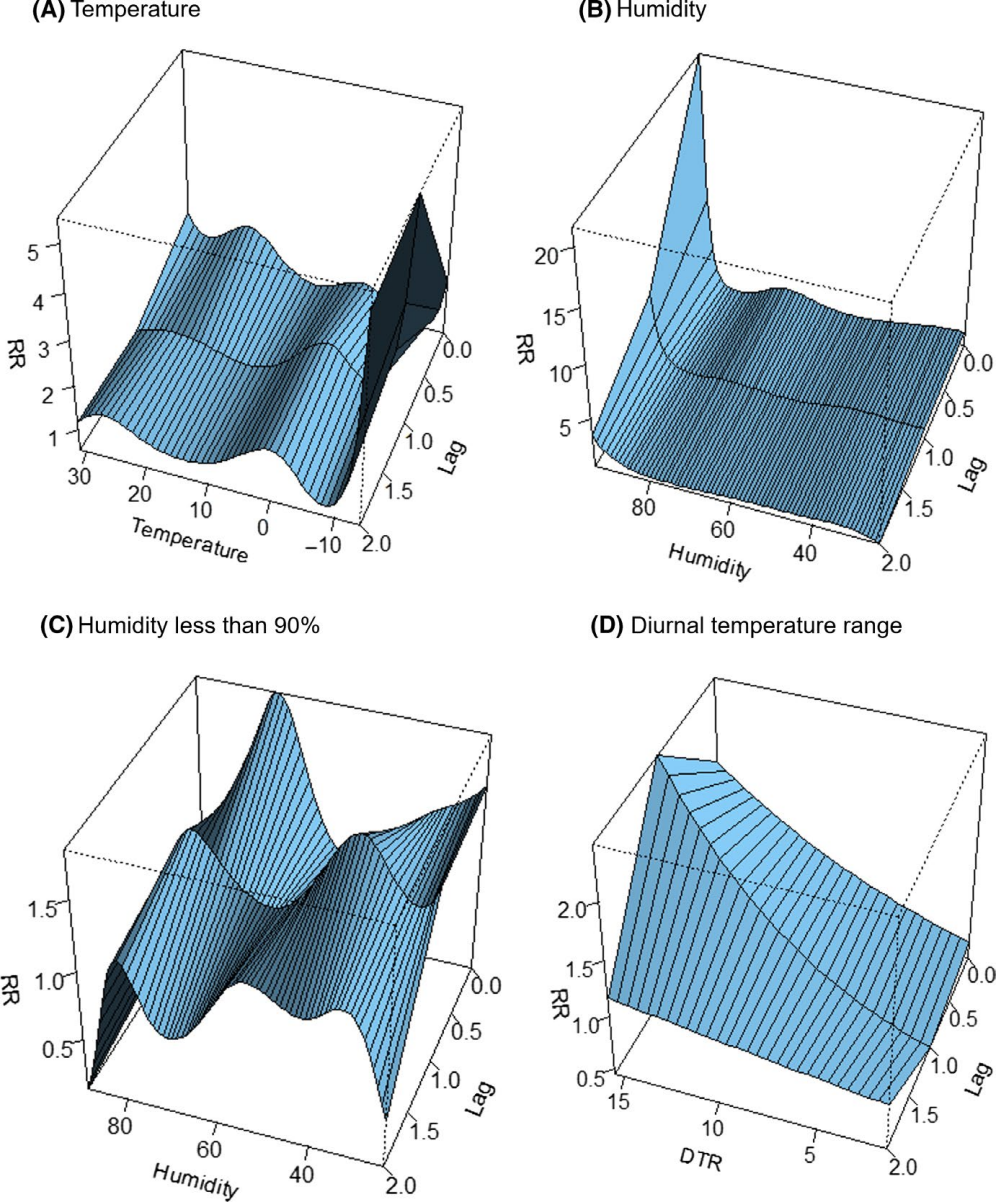
Three‐dimensional plot of relative risk (RR) for temperature (A), humidity (B), and diurnal temperature range (DTR) (C) and lags for influenza incidence

Figure 2 shows the three‐dimensional relationship between weekly meteorological data and influenza cases across 2 lag weeks. Overall, the estimated effects of temperature and humidity on influenza incidence were non‐linear. A visual inspection of the figure suggests that there was increased RR of influenza at low temperatures and a large DTR. The effect of a large DTR decreased over the lag period (Figure 2). With respect to humidity, since data of 6 weeks with over 90% relative humidity showed a high risk of influenza occurrence, the relationship between influenza and humidity lower than 90% was also shown in Figure 2C.

The RR of influenza for specific lag periods (0, 1, and 2 weeks) and specific values of temperature, humidity, and DTR are presented in Table 3. Values of temperature, humidity, and DTR were selected, considering the distribution of each factor. Temperatures lower than the reference value (10°C) were associated with increased influenza incidence across all lag days, and the overall effect was significant only at 0°C (RR: 4.04, 95% CI: 1.25‐13.02) and 5°C (RR: 1.96, 95% CI: 1.09‐3.51), shown in Table 3.

**Table 3:** Relative risks with different values of temperature, humidity, and diurnal temperature range for influenza incidence in Seoul, Republic of Korea

	Value	RR (95% CI)			
		Lag 0	Lag 1 wk	Lag 2 wk	Lag 0‐2 wk (Overall effect)
Temperature (°C)	0	1.26 (0.70, 2.24)	1.90*(1.04, 3.46)	1.70 (0.93, 3.08)	4.04*(1.25, 13.02)
	5	1.05 (0.75, 1.46)	1.36 (0.97, 1.90)	1.38 (0.98, 1.93)	1.96*(1.09, 3.51)
	15	1.48 (0.85, 2.59)	1.04 (0.58, 1.86)	0.95 (0.53, 1.69)	1.46 (0.59, 3.61)
	20	1.98 (0.60, 6.47)	1.23 (0.37, 4.12)	1.13 (0.34, 3.72)	2.76 (0.38, 19.79)
Relative humidity (%)	30	1.74*(1.05, 2.87)	1.48 (0.90, 2.43)	1.07 (0.64, 1.81)	2.76*(1.24, 6.13)
	40	1.25 (0.80, 1.94)	1.65*(1.11, 2.46)	1.14 (0.76, 1.71)	2.34*(1.17, 4.71)
	50	0.85 (0.66, 1.11)	1.25 (0.98, 1.60)	1.06 (0.83, 1.35)	1.13 (0.75, 1.72)
	70	1.90*(1.37, 2.62)	1.23 (0.89, 1.70)	0.83 (0.59, 1.17)	1.93*(1.08, 3.46)
	80	0.84 (0.49, 1.46)	1.12 (0.67, 1.87)	0.87 (0.57, 1.33)	0.83 (0.03, 2.08)
DTR (°C)	2	0.64*(0.46, 0.88)	0.49*(0.35, 0.68)	0.88 (0.62, 1.25)	0.27*(0.15, 0.48)
	5	0.80*(0.68, 0.94)	0.70*(0.59, 0.82)	0.94 (0.79, 1.12)	0.52*(0.39, 0.70)
	11	1.25*(1.06, 1.47)	1.44*(1.22, 1.69)	1.07 (0.89, 1.27)	1.92*(1.44, 2.56)
	14	1.57*(1.13, 2.17)	2.06*(1.48, 2.87)	1.14 (0.80, 1.62)	3.67*(2.06, 6.54)

Note

For temperature and DTR, 10 and 8°C, which are close to mean values, were used as the reference values, respectively, to calculate RR. For relative humidity, 60% was used as reference.

Abbreviations: CI, confidence interval; DTR, diurnal temperature range; RR, relative risk.

* *P* < .05.

Low humidity (30%) showed increased risk for the current (RR: 1.1, 95% CI: 1.05 − 1.16) periods and overall effect (RR: 2.76, 95% CI: 1.24 − 6.13). Lower relative humidity (40%) than the reference (60%) also showed higher influenza risk (RR: 2.34, 95% CI: 1.17 − 4.71). Although 80% relative humidity showed lower risk of influenza, 70% showed overall increased risk of influenza as compared with 60% (RR: 1.93, 95% CI: 1.08 − 3.46).

The effect of DTR on influenza was significant over short‐term periods, such as lag 0 days, and 1‐2 weeks lag period. For current, lag one, and lag 2 weeks, all DTR values had a significant effect on influenza incidence. A 3°C increase or decrease in DTR was associated with an approximately 30% change in influenza incidence. The overall effect of lower DTR (RR: 0.27, 95% CI: 0.15‐0.48 for DTR 2°C; and RR: 0.52, 95% CI: 0.39‐0.70 for DTR 5°C) as compared with the reference showed decreased risk and that of higher DTR showed increased risk (RR: 1.92, 95% CI: 1.44‐2.56 for DTR 11°C; and RR: 3.67, 95% CI: 2.06‐6.54 for DTR 14°C).

We changed the lag periods for meteorological data, and the effect of these factors and trends were not substantially changed, and the effect of DTR was significant in all models. Changes in the degrees of freedom for seasonal control showed similar results (Figure S1). A residual plot was also created, to check the assumption of analysis (Table S2).

## 4 DISCUSSION

Studies conducted in temperate regions, such as that of the Republic of Korea, have yielded contrasting results. Whereas several studies have failed to find an effect of temperature^9^ or humidity,^10^, ^17^ other studies have reported an association between temperature and humidity and the incidence of influenza. Sundell et al^36^ showed that influenza was associated with low temperatures. In the present study, we found that low temperatures were associated with a high incidence of influenza in a temperate region.

Tamerius et al^7^ reported that cold‐dry and humid‐rainy conditions were associated with seasonal influenza epidemics in temperate and tropical areas. The result of the present study also showed a high risk of influenza with high (70%) or lower (<40%) humidity. An increased risk influenza in high humidity higher than 90% might be contributed by small number of cases, which was only 6 weeks. Despite two previous studies reporting that influenza activity was negatively associated with absolute humidity in temperate regions,^15^, ^37^ another study in Japan showed an increased influenza incidence in high humidity.^16^ Further studies of the association between humidity and influenza incidence in temperate regions are needed.

Even though previous studies have reported the association between DTR and mortality^20^, ^22^ and respiratory disease,^21^, ^24^ studies investigating the effect of DTR on influenza are rare. One Chinese study reported that DTR was significantly related to influenza seasonality in dry periods but not in humid periods.^25^ In another study conducted in France, the effect of daily temperature variation on seasonal influenza was not significant.^26^ The results of that study showed that high DTR was positively associated with influenza incidence even if the temperatur**e** is same.

We found that temperature and humidity had a short lagged effect, which was limited in one week, on influenza diagnosis. Tsuchihashi et al^10^ reported that lower temperatures during the 8 days prior to clinic visits affected influenza infection, and a Chinese study reported a longer incubation period of 13 days prior to disease onset.^17^ Another Chinese study investigating the impact of temperature on mortality reported that the effects of extremely high temperatures generally persisted for 3 days, whereas the risk with extremely low temperatures could persist for 21 days.^34^ One study conducted in a temperate region used a lag of 1 week to examine the effects of climate factors on seasonal influenza.^26^ Further research assessing the lagged effects of temperature and humidity on health is needed.

During 2010‐2016, two peaks of influenza epidemics occurred in 2010, 2012, 2015, and 2016. Influenza A and B are common in Korea. When peaks of influenza A and B differ, the influenza epidemic pattern shows two peaks.^38^ For example, in early 2012, the first peak was caused by influenza A and the second was owing to influenza B. In 2011, 2013, and 2014, there was one influenza peak. The reason for the presence of only one peak was that the influenza epidemic was only caused by influenza A in 2011 whereas in 2013 and 2014, outbreaks of influenza A and B occurred during similar periods.^38^

The range of climate factors affecting influenza activity may differ by virus strain. Human infection with avian influenza strain H7N9 has been reported to correlate with temperature (4‐14°C) and relative humidity (65%–95%) whereas infection with H5N1 is correlated with temperature and atmospheric pressure (980‐1025 kPa).^39^ There is clearly a need to investigate the rates of influenza incidence according to both climate and virus strain.

One limitation of this study is that weekly data rather than daily data were used; daily influenza incidence can reflect the change in daily weather better than weekly influenza incidence. However, this study was focused on the trend of influenza incidence and weather over a long period (ie, 6 years) rather than minor changes over a short duration. Thus, the present results provide information about the association between weather and influenza incidence.

Apart from temperature and humidity, previous studies have reported that seasonal changes in individual behavior and family and social structures^5^ might be associated with the incidence of influenza. In addition, a recent study showed an association between a decline in vaccination coverage and an increase in influenza incidence.^40^ We could not control for those factors in this study because of limited data. Social and behavioral factors should be considered in future studies.

## CONFLICT OF INTEREST

The author declares that they have no competing interests.

## RESEARCH INVOLVING HUMAN PARTICIPANTS AND/OR ANIMALS

The study did not include human participants or animals.
