# Supplementary figures and images for: Effects of temperature, humidity, and diurnal temperature range on influenza incidence in a temperate region
Source: Influenza Other Respir Viruses. 2019 Oct 21;14(1):11–8. doi: 10.1111/irv.12682 (PMC6928031; doi:10.1111/irv.12682)

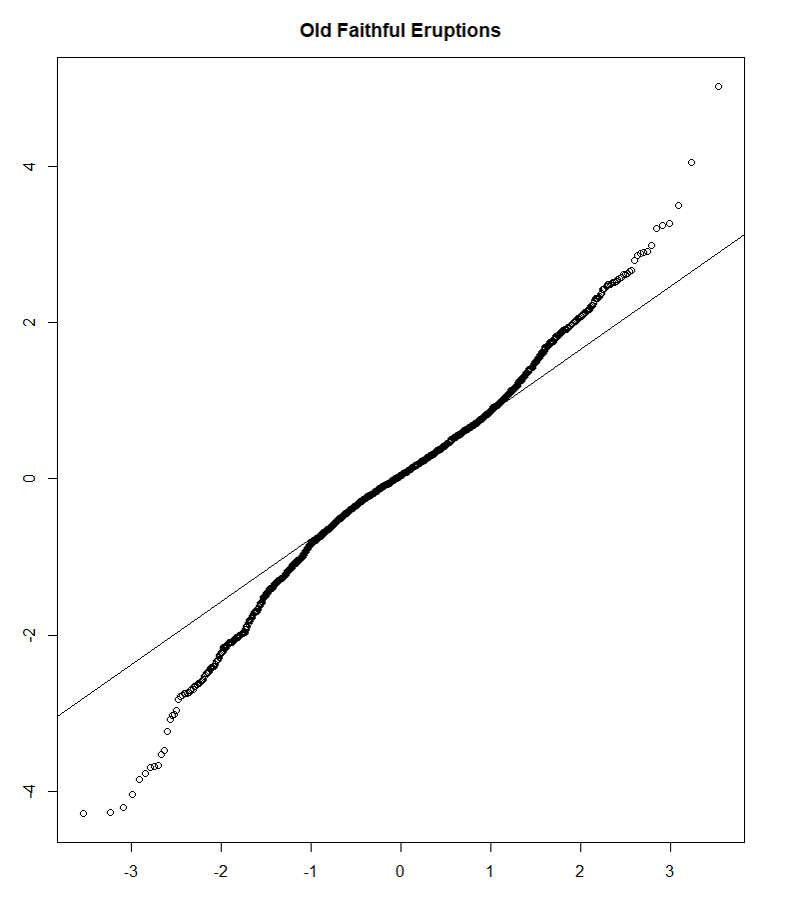

Supplement: Supplementary file 1 [file IRV-14-11-s001.png]
