# Supplementary material for: Effects of temperature, humidity, and diurnal temperature range on influenza incidence in a temperate region
Source: Influenza Other Respir Viruses. 2019 Oct 21;14(1):11–8. doi: 10.1111/irv.12682 (PMC6928031; doi:10.1111/irv.12682)
Supplement: Supplementary file 2 [file IRV-14-11-s002.docx]

Supplementary File 2. Overall effect of temperature, humidity, and diurnal temperature range with various model choices, as a sensitivity analysis.

|  |  | Overall effect (95% CI) | | | | |
| --- | --- | --- | --- | --- | --- | --- |
|  |  | Main model | lag period: one week | lag period: three weeks | 5 df/year for seasonal control | 9 df/year for seasonal control |
| Temperature (℃) | 0 | **4.04^*^(1.25, 13.02)** | **1.48 (0.59, 3.72)** | **4.06^*^(1.06, 15.60)** | **3.20^*^(1.10, 9.29)** | **2.68 (0.82, 8.76)** |
|  | 5 | **1.96^*^(1.09, 3.51)** | **0.92 (0.50, 1.71)** | **1.74 (0.91, 3.31)** | **1.86^*^(1.06, 3.25)** | **1.74 (0.95, 3.16)** |
|  | 15 | **1.46 (0.59, 3.61)** | **1.20 (0.59, 2.47)** | **2.72^*^(1.05, 7.05)** | **1.17 (0.60, 2.29)** | **1.22 (0.50, 2.99)** |
|  | 20 | **2.76 (0.38, 19.79)** | **1.34 (0.21, 8.52)** | **10.65^*^(1.32, 85.96)** | **1.51 (0.33, 6.88)** | **2.03 (0.27, 15.02)** |
| Relative humidity (%) | 30 | **2.76^*^(1.24, 6.13)** | **1.27 (0.56, 2.85)** | **2.10 (0.86, 5.13)** | **2.65^*^(1.20, 5.86)** | **2.04 (0.98, 5.09)** |
|  | 40 | **2.34^*^(1.17, 4.71)** | **1.60 (0.90, 2.86)** | **1.93 (0.87, 4.24)** | **2.23^*^(1.11, 4.48)** | **2.00 (0.98, 4.08)** |
|  | 50 | **1.13 (0.75, 1.72)** | **0.78 (0.52, 1.16)** | **1.09 (0.68, 1.75)** | **1.10 (0.73, 1.66)** | **1.05 (0.69, 1.60)** |
|  | 70 | **1.93^*^(1.08, 3.46)** | **1.87^*^(1.24, 2.82)** | **2.08^*^(1.03, 4.21)** | **1.87^*^(1.05, 3.34)** | **1.64 (0.91, 2.96)** |
|  | 80 | **0.83 (0.03, 2.08)** | **0.87 (0.40, 1.93)** | **1.01 (0.35, 2.93)** | **0.72 (0.30, 1.75)** | **0.59 (0.23, 1.53)** |
| DTR (℃) | 2 | **0.27^*^(0.15, 0.48)** | **0.40^*^(0.24, 0.66)** | **0.40^*^(0.20, 0.77)** | **0.26^*^(0.15, 0.47)** | **0.26^*^(0.14, 0.46)** |
|  | 5 | **0.52^*^(0.39, 0.70)** | **0.63^*^(0.49, 0.81)** | **0.63^*^(0.45, 0.88)** | **0.51^*^(0.39, 0.68)** | **0.51^*^(0.38, 0.68)** |
|  | 11 | **1.92^*^(1.44, 2.56)** | **1.58^*^(1.23, 2.04)** | **1.59^*^(1.14, 2.22)** | **1.94^*^(1.46, 2.58)** | **1.96^*^(1.47, 2.63)** |
|  | 14 | **3.67^*^(2.06, 6.54)** | **2.50^*^(1.51, 4.15)** | **2.52^*^(1.30, 4.91)** | **3.77^*^(2.13, 6.68)** | **3.86^*^(2.16, 6.90)** |

^*^: p<0.05. For temperature and DTR, 10°C and 8°C, which are close to the mean values, were used as the reference values, respectively, to calculate relative risk. For relative humidity, 60% was used as reference.
